# Supplementary material for: Synthesis and Antimicrobial Activity of the Pathogenic E. coli Strains of p-Quinols: Additive Effects of Copper-Catalyzed Addition of Aryl Boronic Acid to Benzoquinones
Source: Int J Mol Sci. 2023 Jan 13;24(2):1623. doi: 10.3390/ijms24021623 (PMC9862949; doi:10.3390/ijms24021623)
Supplement: Supplementary file 1 [file ijms-24-01623-s001.zip › ijms-2135255-SI.pdf]

Supplementary materials

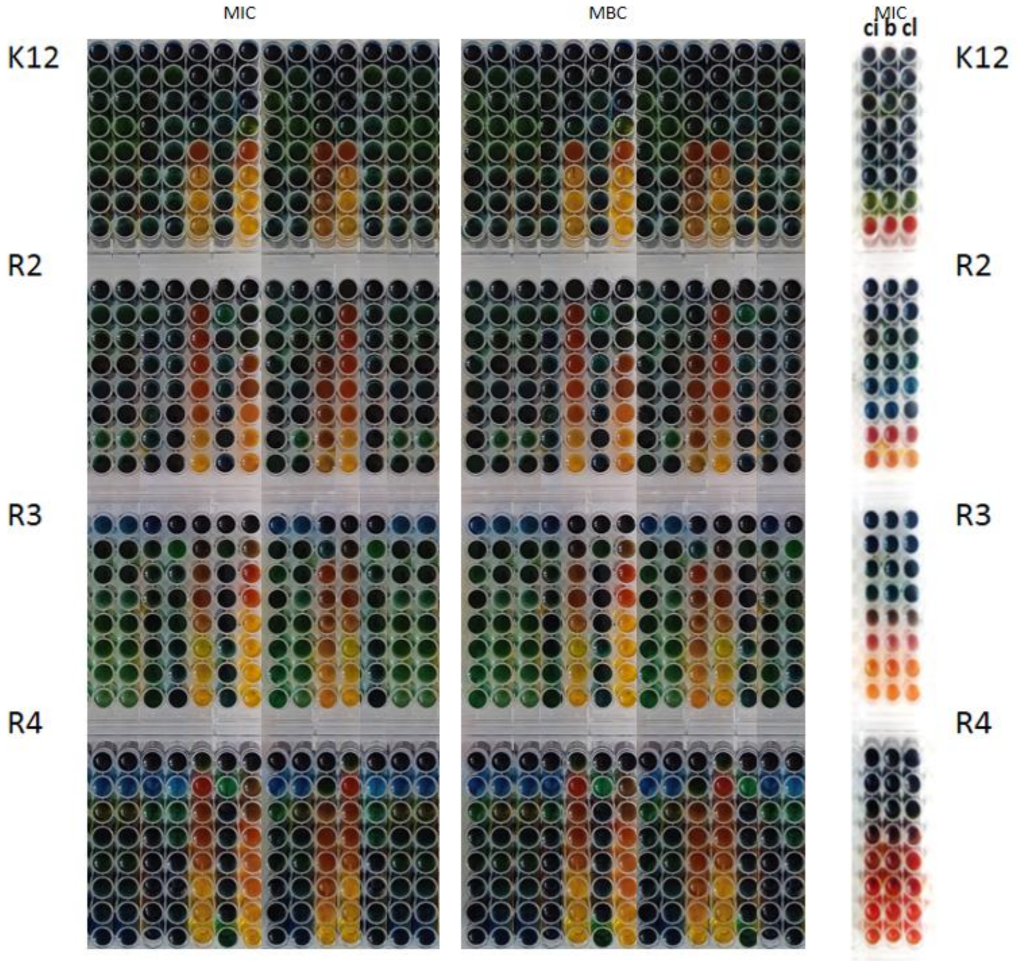

Figure S1. Examples of MIC and MBC on microplates with different concentration of studied compounds (μg/mL^{-1}). Resazurin was added as an indicator of microbial growth with K12, R2, R3, and R4 strains with tested compounds, as described in Figure 4 and 5 . Additionally, examples of MIC with different strains K12, R2, R3, and R4 of studied antibiotics with ciprofloxacin (ci), bleomycin (b), and cloxacillin (cl) in (μg/mL^{-1}).

Panel A

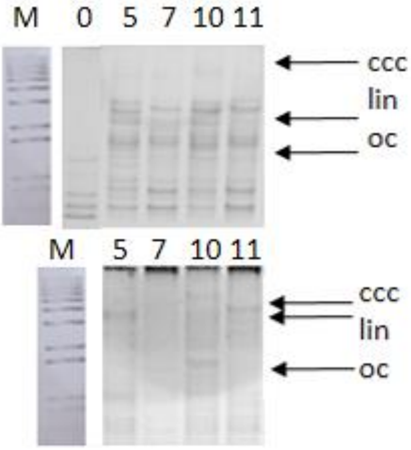

Panel B

Figure S2. An example of an agarose gel electrophoresis separation of isolated plasmids DNA on R4 strains modified with selected coumarin derivatives (Panel A) from 4 selected compounds, as shown in Figure 3, and digested with repair Fpg protein (Panel B). M = marker.

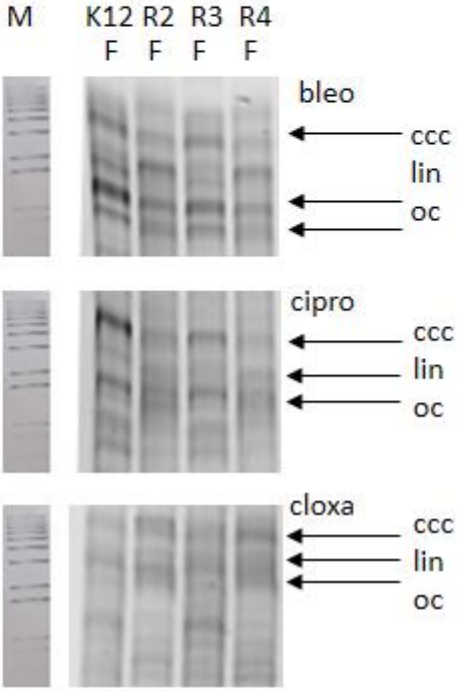

Figure S3. Example of an agarose gel electrophoresis separation of isolated plasmids DNA from K12 and R4 strains modified with antibiotics: bleomycin, ciprofloxacin, and cloxacillin digested (or not) with repair enzymes Fpg. M = marker.

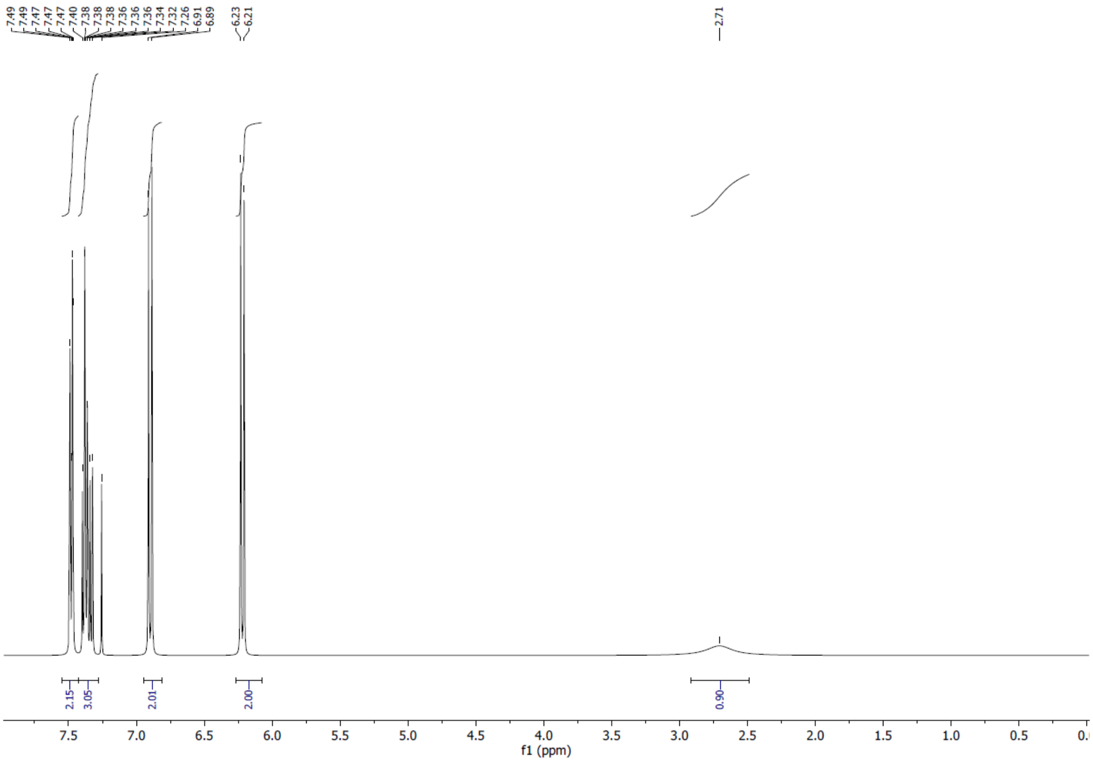

Figure S4. ^{1}H NMR spectra (400 MHz, $CDCl_3$) of 4-hydroxy-4-phenyl-cyclohexa-2,5-dienone (**1**)

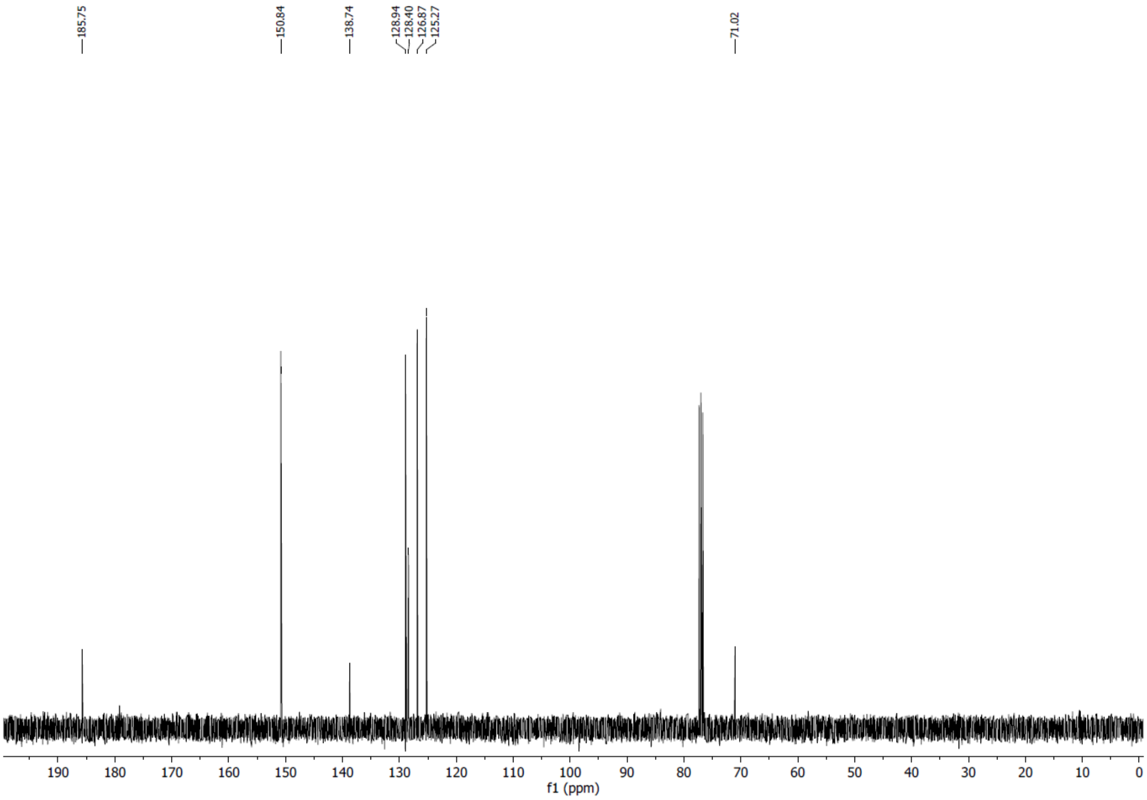

Figure S5. ^{13}C NMR spectra (100 MHz, $CDCl_3$) of 4-hydroxy-4-phenyl-cyclohexa-2,5-dienone (**1**)

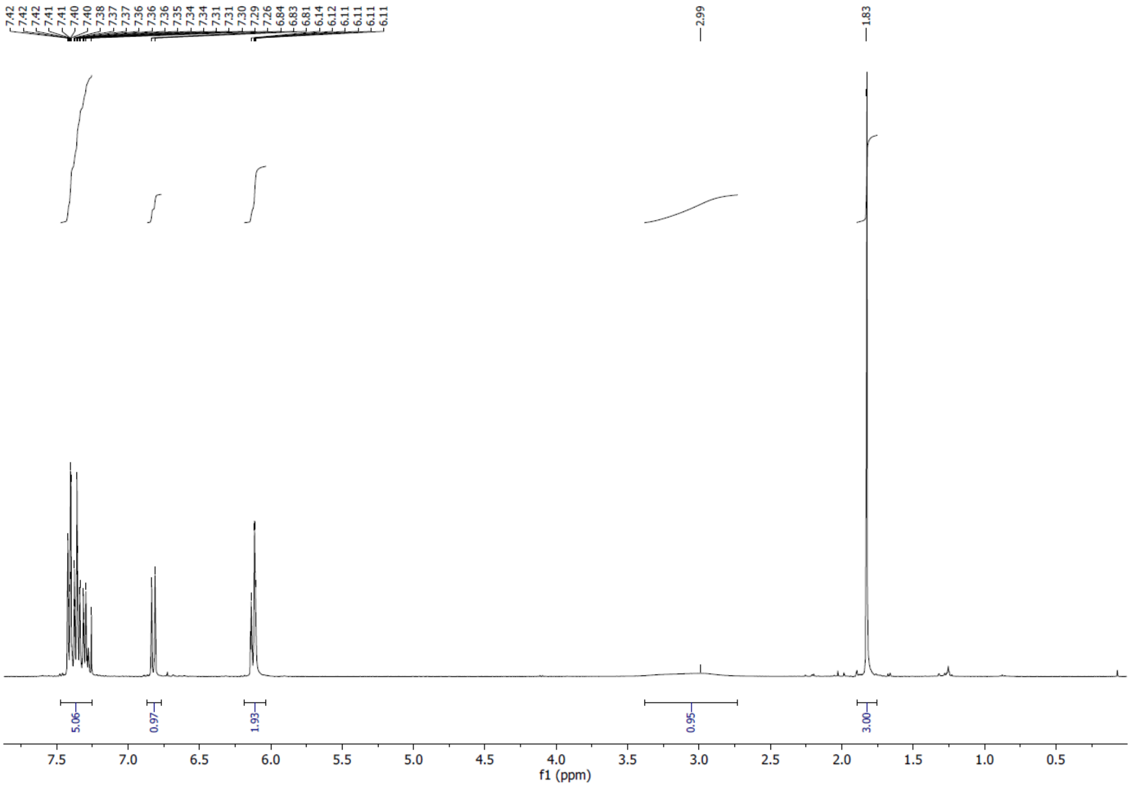

Figure S6. ^{1}H NMR spectra (400 MHz, $CDCl_3$) of 4-hydroxy-2-methyl-4-phenyl-2,5-cyclohexadienone (**2**)

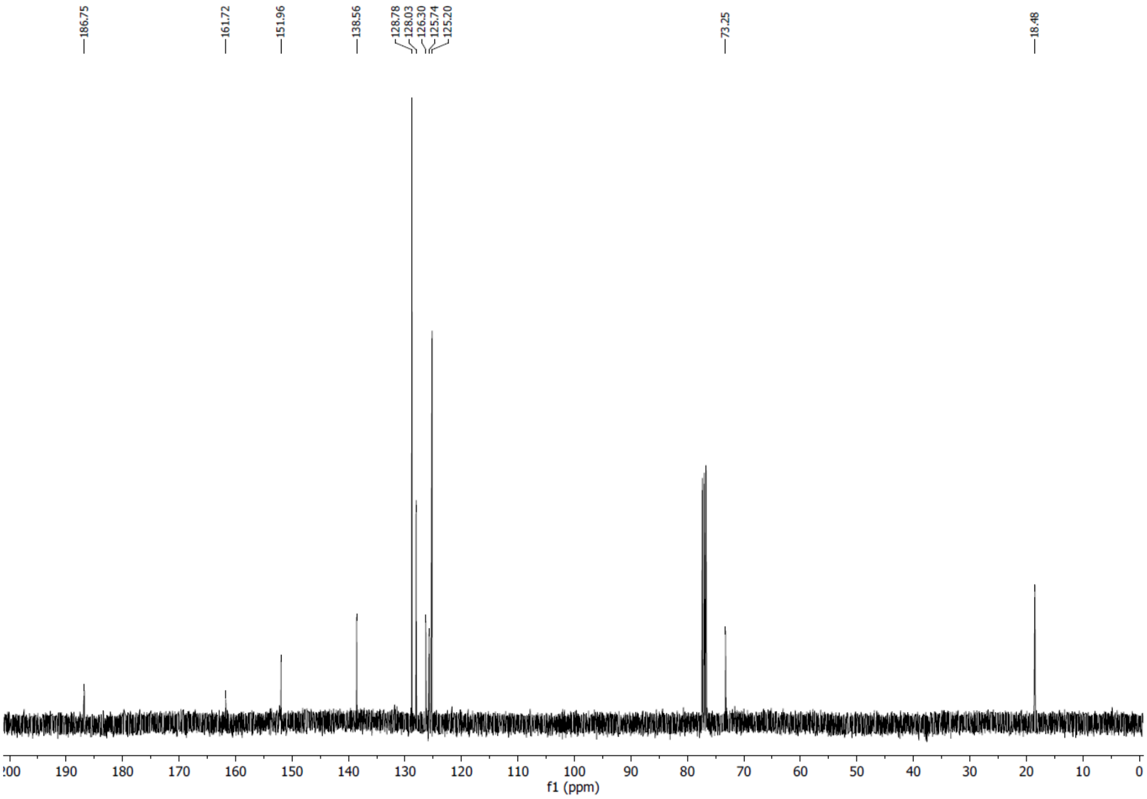

Figure S7. ^{13}C NMR spectra (100 MHz, $CDCl_3$) of 4-hydroxy-2-methyl-4-phenyl-2,5-cyclohexadienone (**2**)

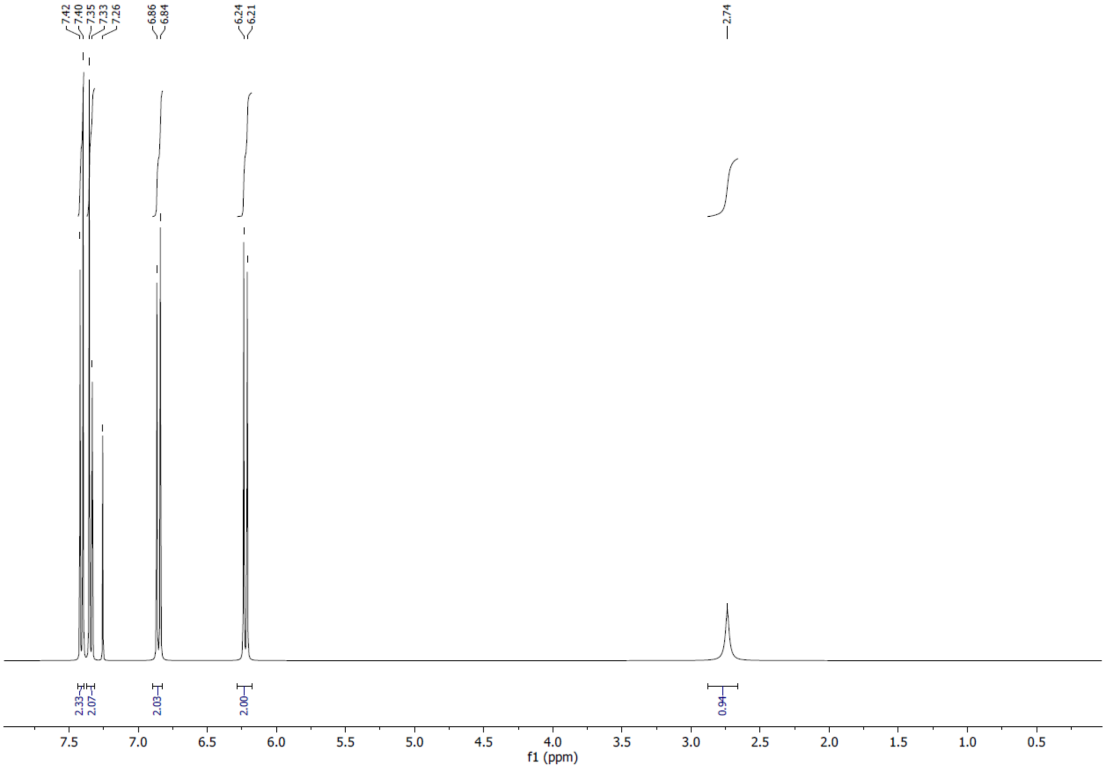

Figure S8. ^{1}H NMR spectra (400 MHz, $CDCl_3$) of 4-hydroxy-4-(4'-chloro)-phenyl-cyclohexa-2,5-dienone (3)

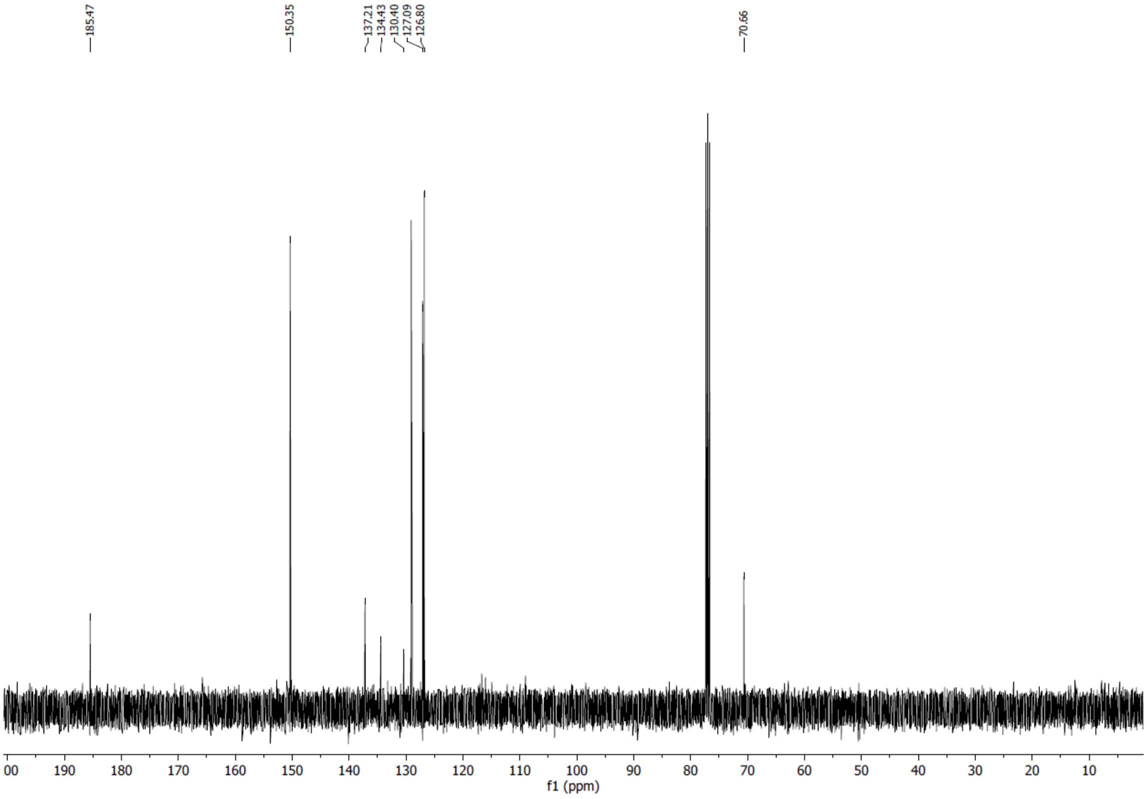

Figure S9. ^{13}C NMR spectra (100 MHz, $CDCl_3$) of 4-hydroxy-4-(4'-chloro)-phenyl-cyclohexa-2,5-dienone (3)

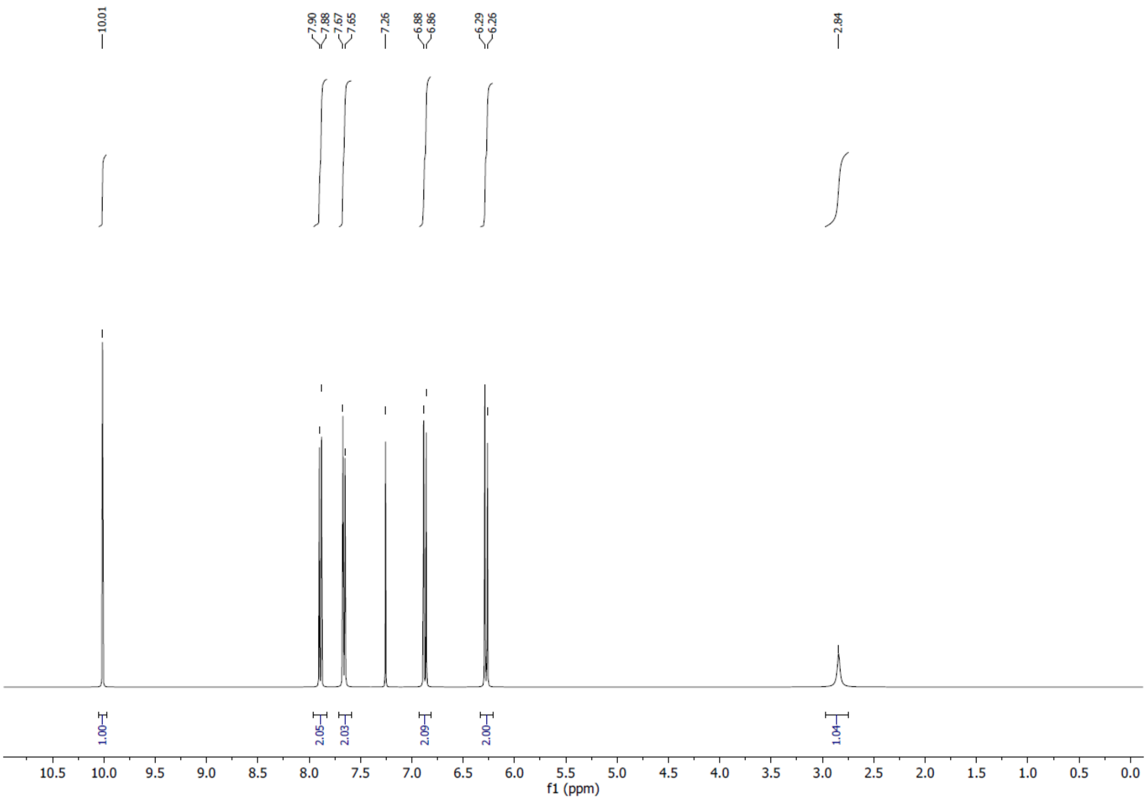

Figure S10. ^{1}H NMR spectra (400 MHz, $CDCl_3$) of 4-hydroxy-4-(4'-formyl)-phenyl-cyclohexa-2,5-dienone (4)

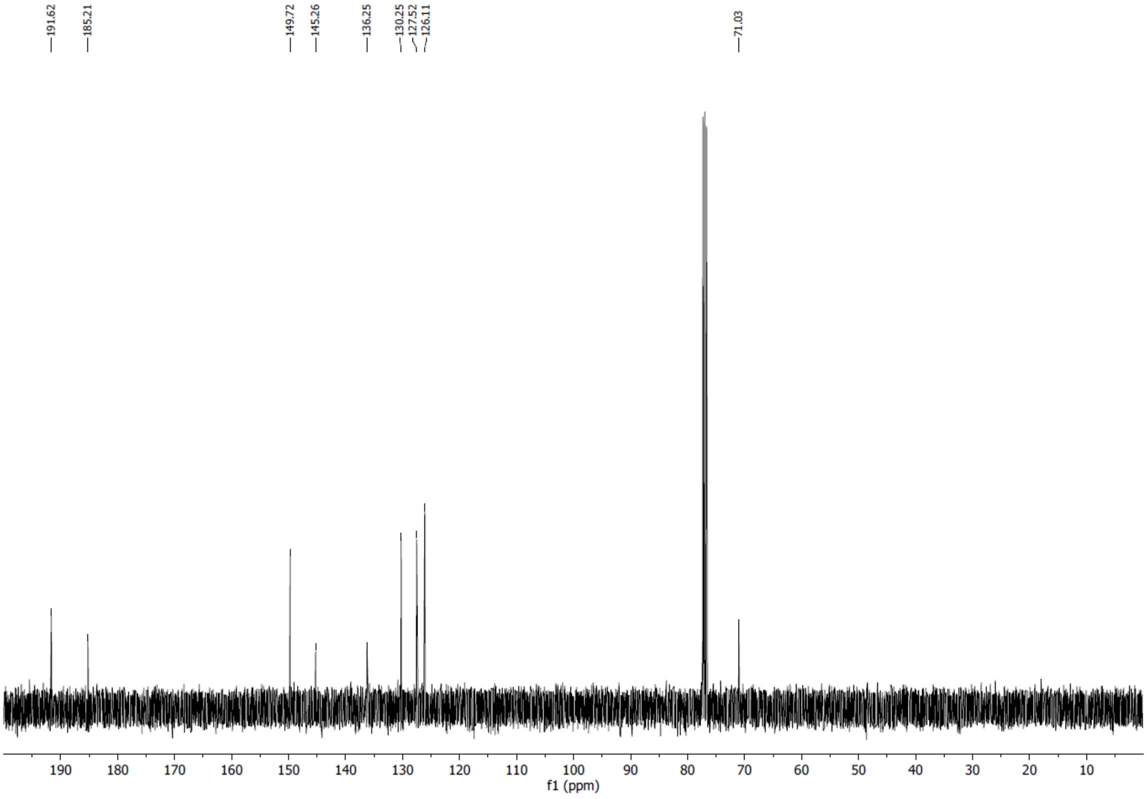

Figure S11. ^{13}C NMR spectra (100 MHz, $CDCl_3$) of 4-hydroxy-4-(4'-formyl)-phenyl-cyclohexa-2,5-dienone (4)

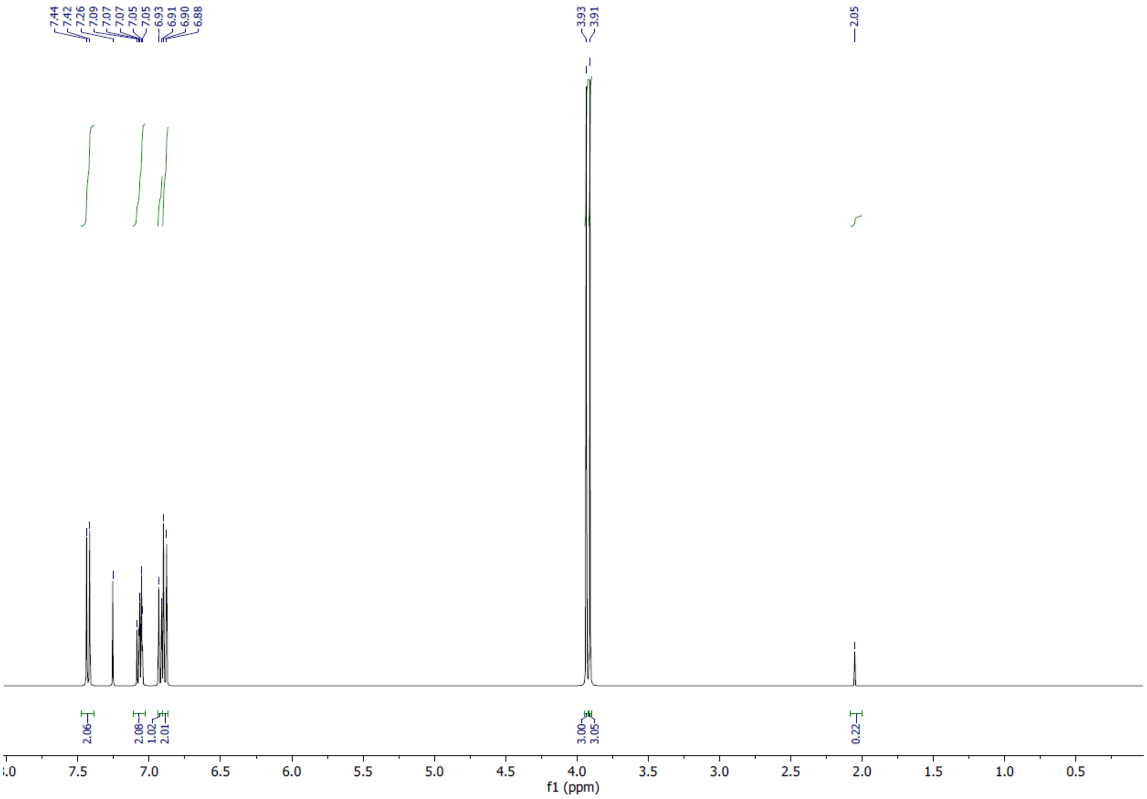

Figure S12. ^{1}H NMR spectra (400 MHz, $CDCl_3$) of 4-(3,4-dimethoxyphenyl)-4-hydroxycyclohexa-2,5-dien-1-one (5)

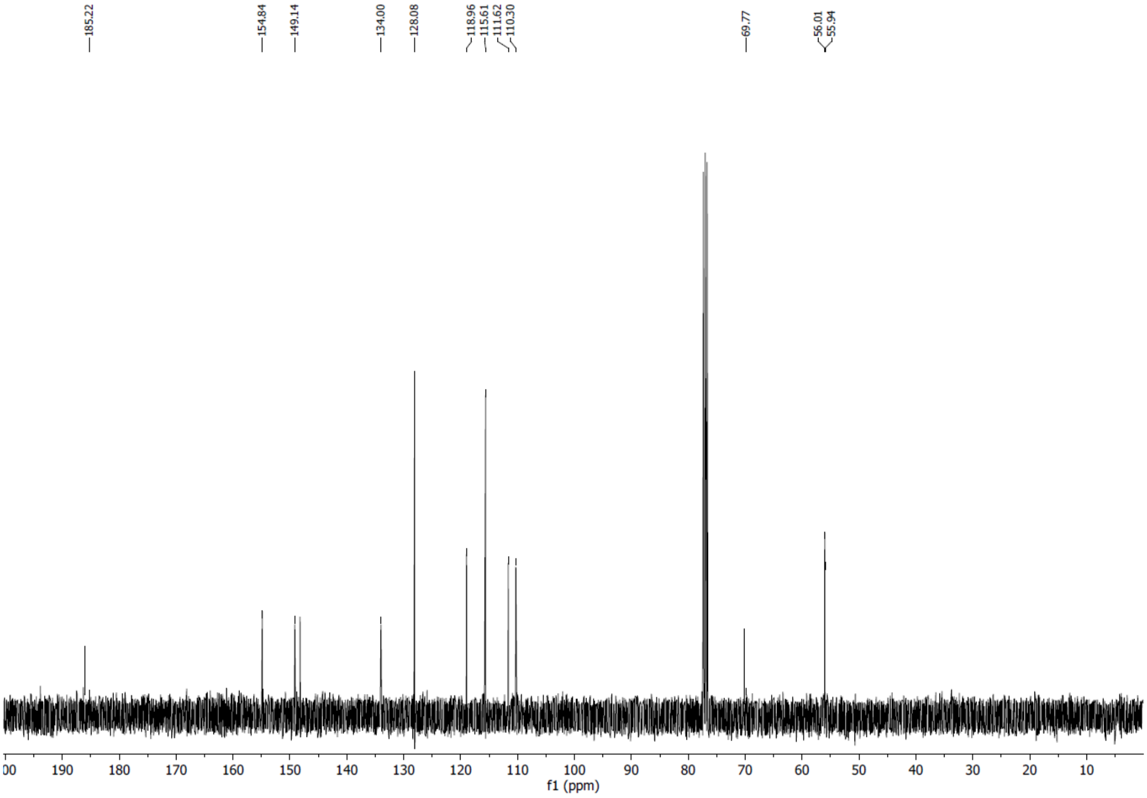

Figure S13. ^{13}C NMR spectra (100 MHz, $CDCl_3$) of 4-(3,4-dimethoxyphenyl)-4-hydroxycyclohexa-2,5-dien-1-one (5)

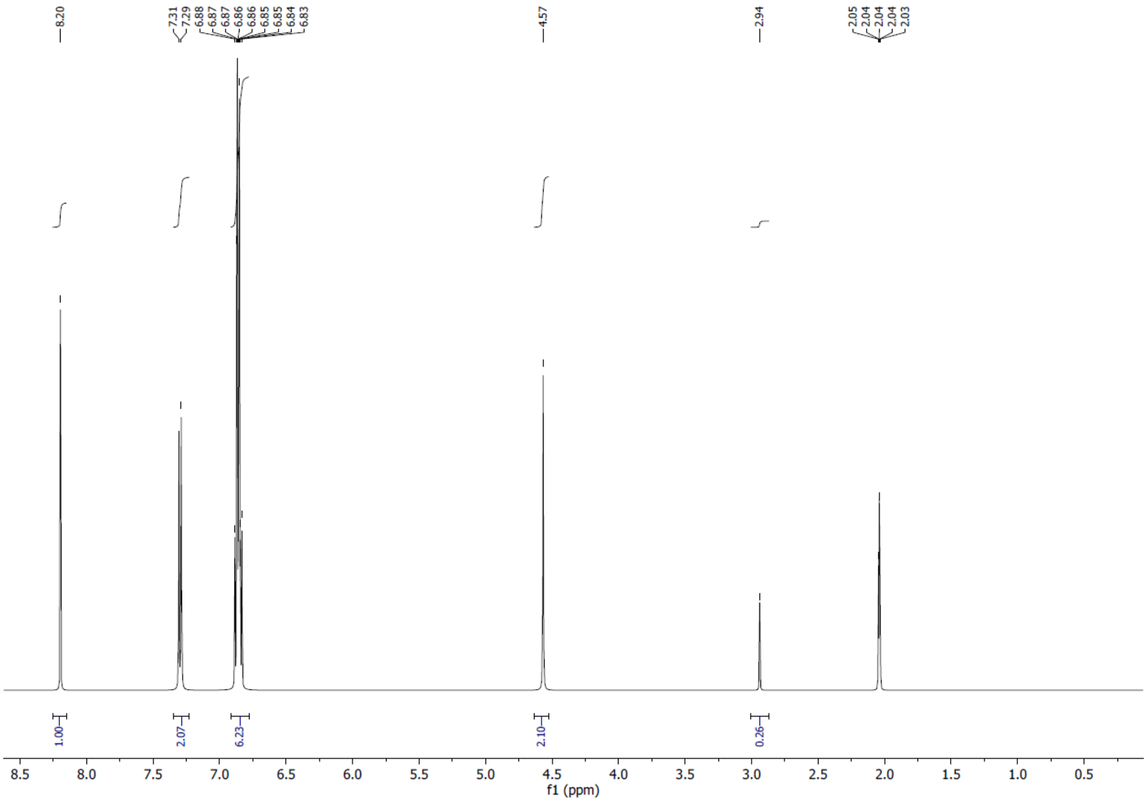

Figure S14. ^{1}H NMR spectra (400 MHz, $CDCl_3$) of 4-hydroxy-4-(4'-hydroxymethylphenyl) phenyl-cyclohexa-2,5-dienone (6)

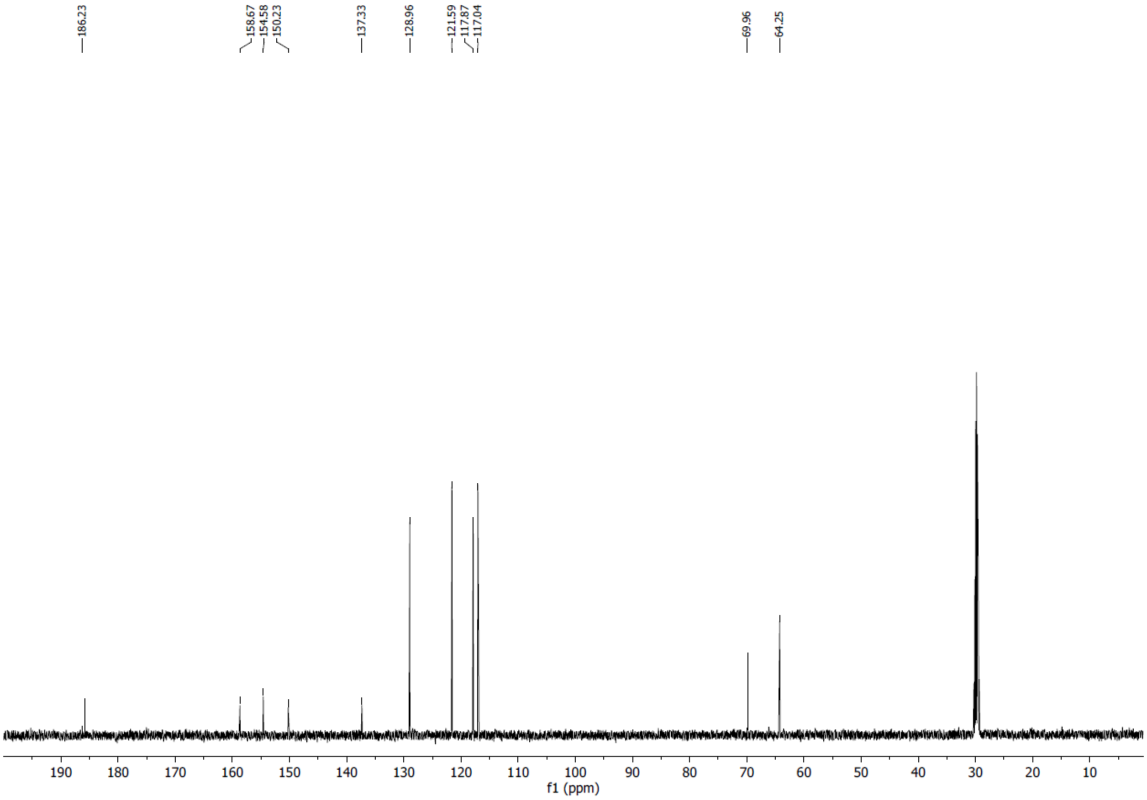

Figure S15. ^{13}C NMR spectra (100 MHz, $CDCl_3$) of 4-hydroxy-4-(4'-hydroxymethylphenyl) phenyl-cyclohexa-2,5-dienone (6)

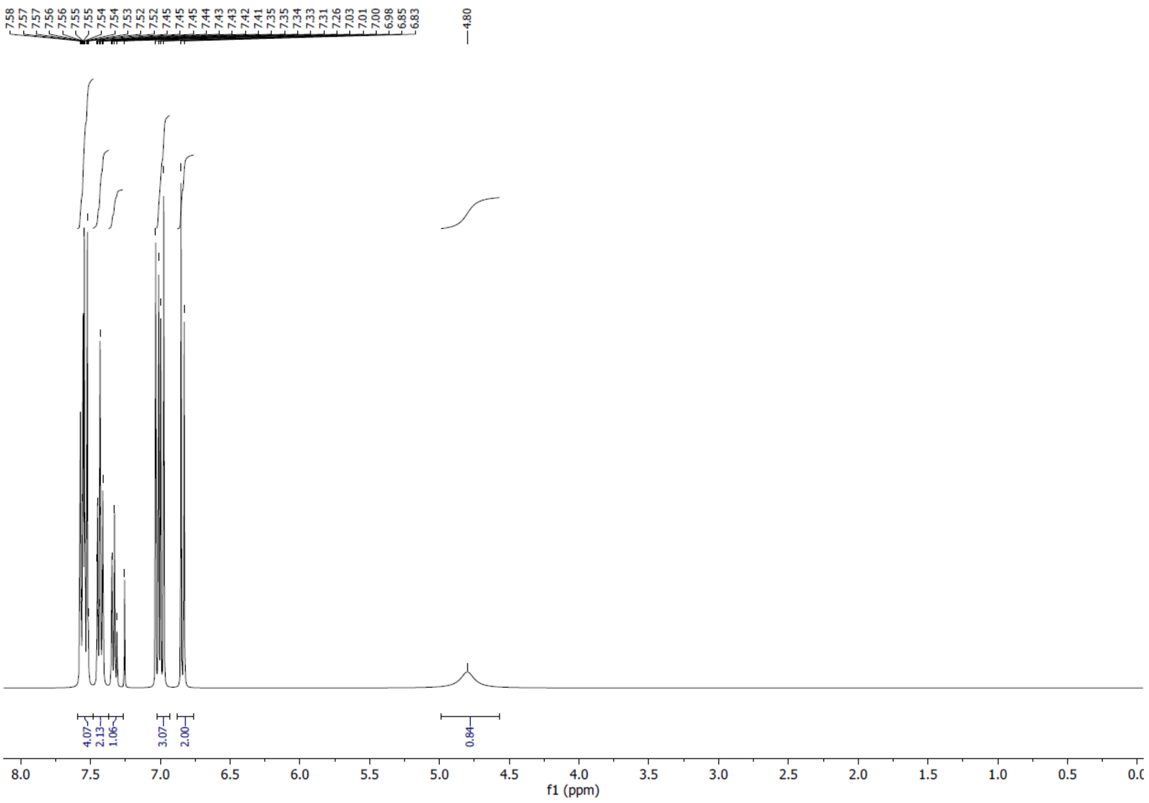

Figure S16. ^{1}H NMR spectra (400 MHz, $CDCl_3$) of 4-hydroxy-4-(4'-biphenyl) phenyl-cyclohexa-2,5-dienone (7)

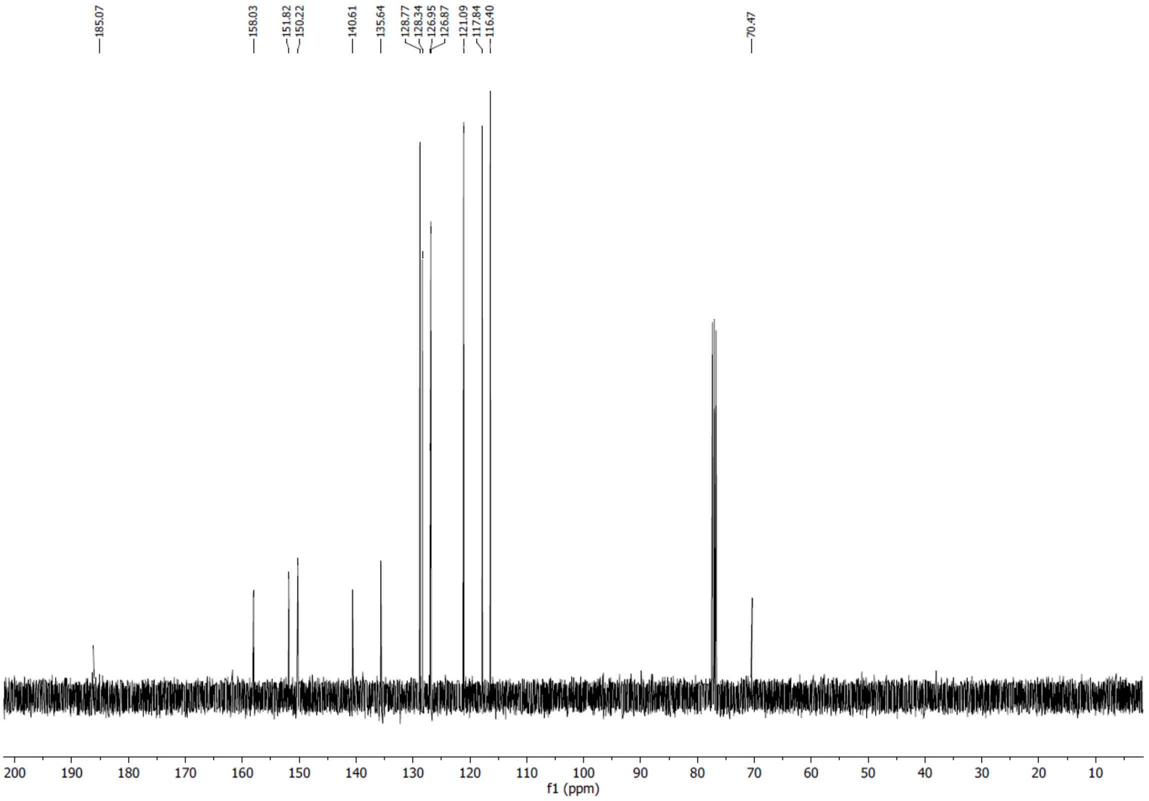

Figure S17. ^{13}C NMR spectra (100 MHz, $CDCl_3$) of 4-hydroxy-4-(4'-biphenyl) phenyl-cyclohexa-2,5-dienone (7)

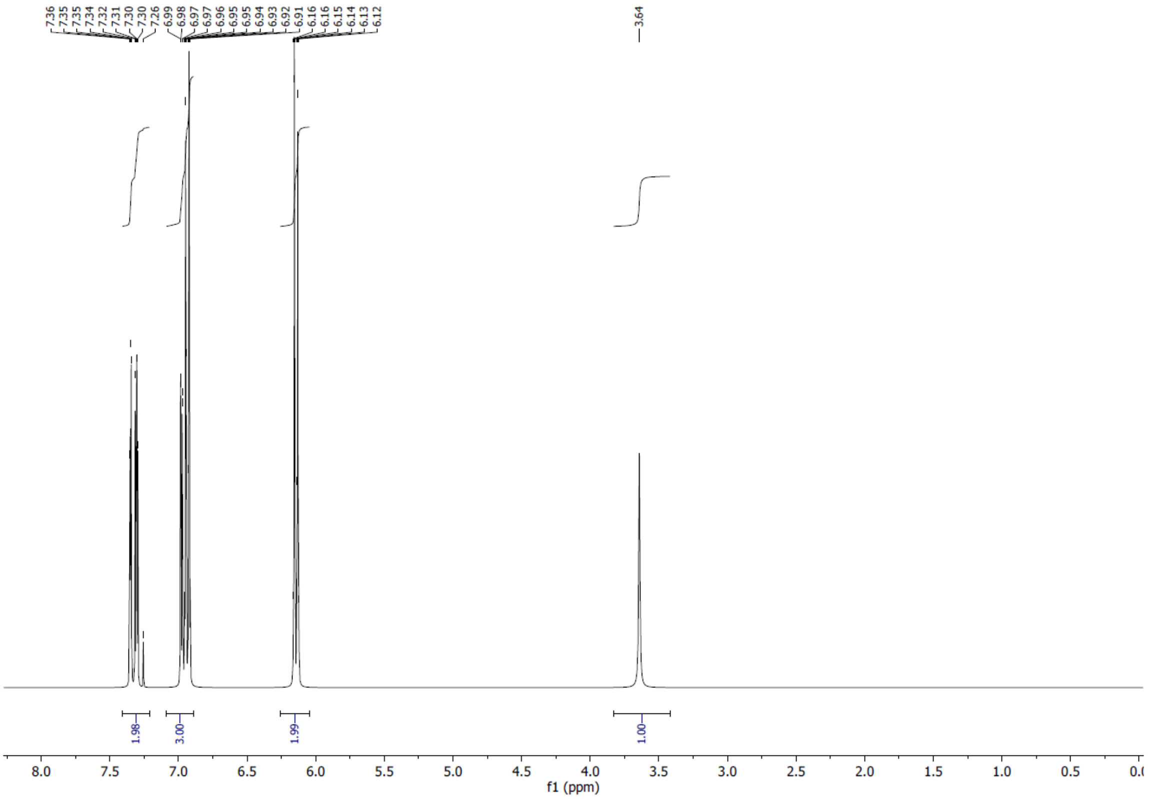

Figure S18. ^{1}H NMR spectra (400 MHz, $CDCl_3$) of 4-hydroxy-4-(thiophen-3-yl) phenyl-cyclohexa-2,5-dienone (8)

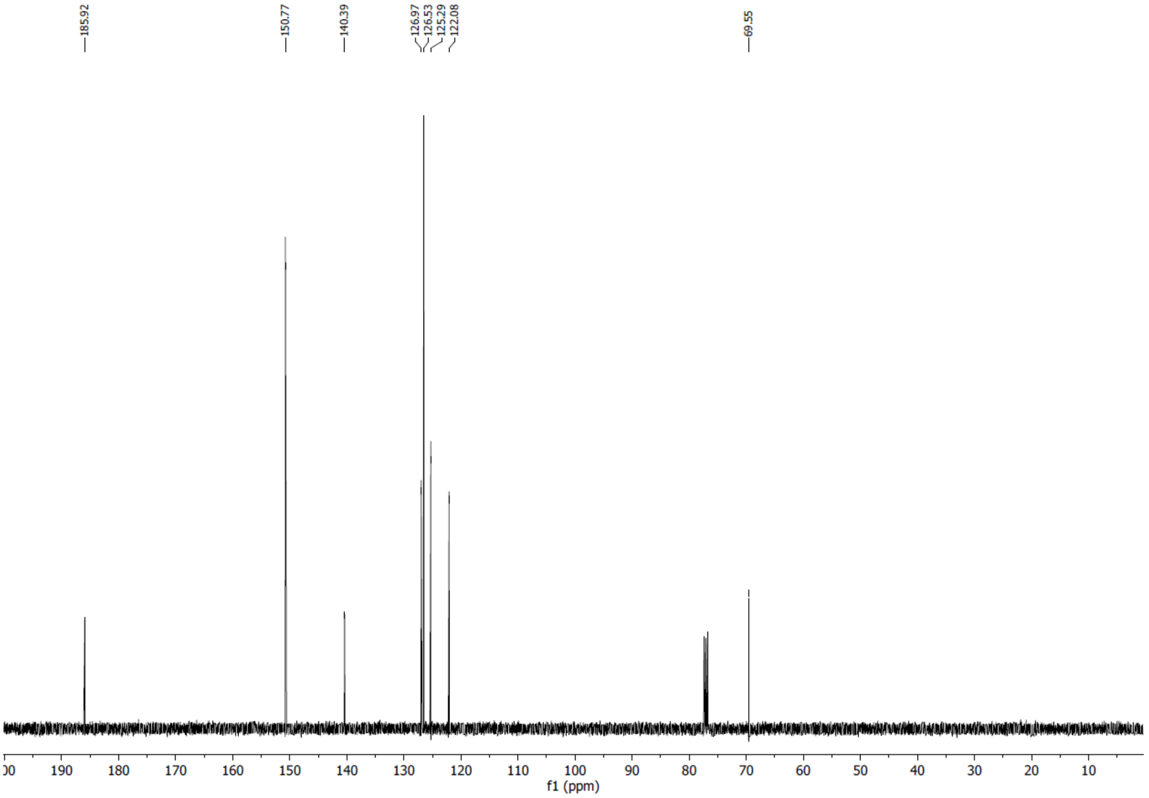

Figure S19. ^{13}C NMR spectra (100 MHz, $CDCl_3$) of 4-hydroxy-4-(thiophen-3-yl) phenyl-cyclohexa-2,5-dienone (8)

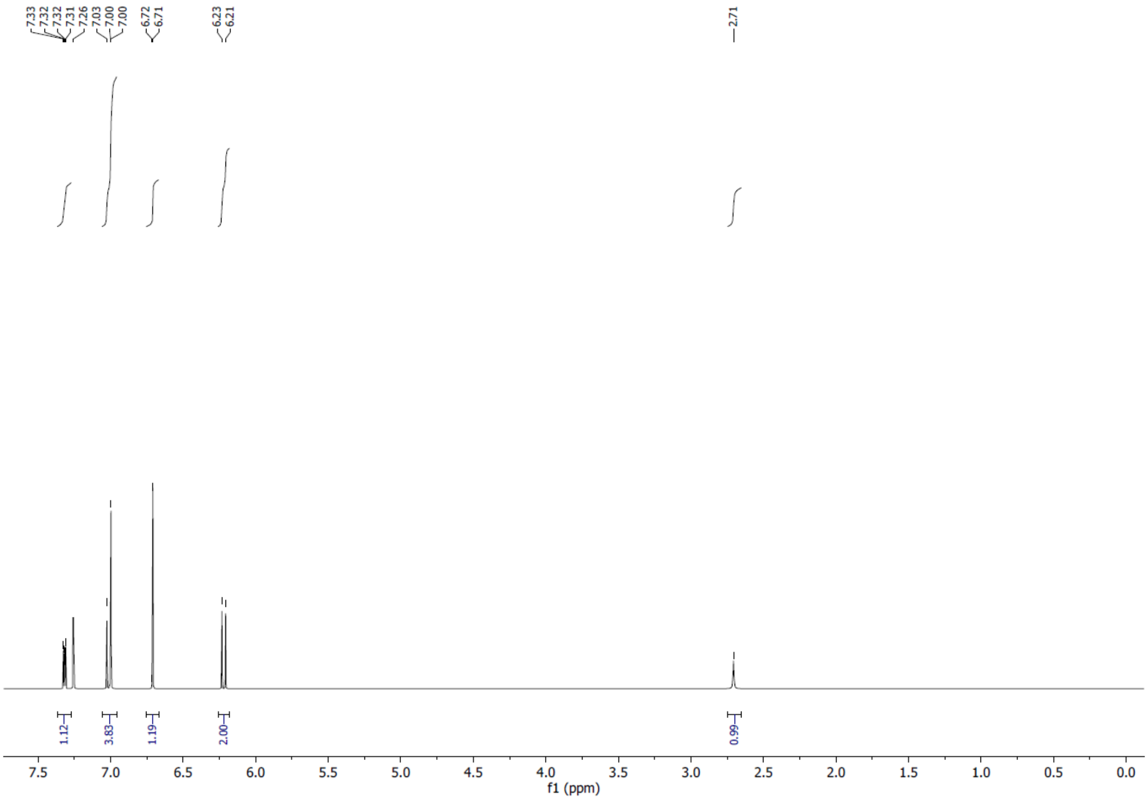

Figure S20. ^{1}H NMR spectra (400 MHz, $CDCl_3$) of 4-hydroxy-4-(furan-3-yl) phenyl-cyclohexa-2,5-dienone (9)

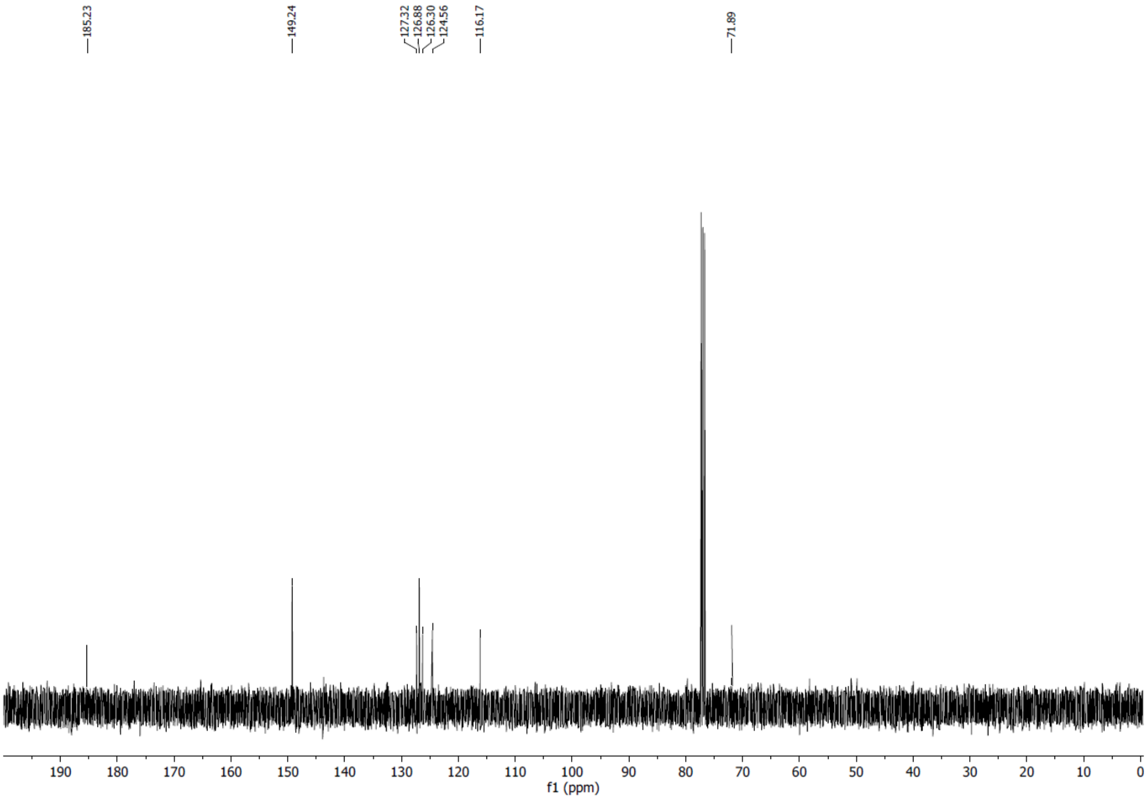

Figure S21. ^{13}C NMR spectra (100 MHz, $CDCl_3$) of 4-hydroxy-4-(furan-3-yl) phenyl-cyclohexa-2,5-dienone (9)

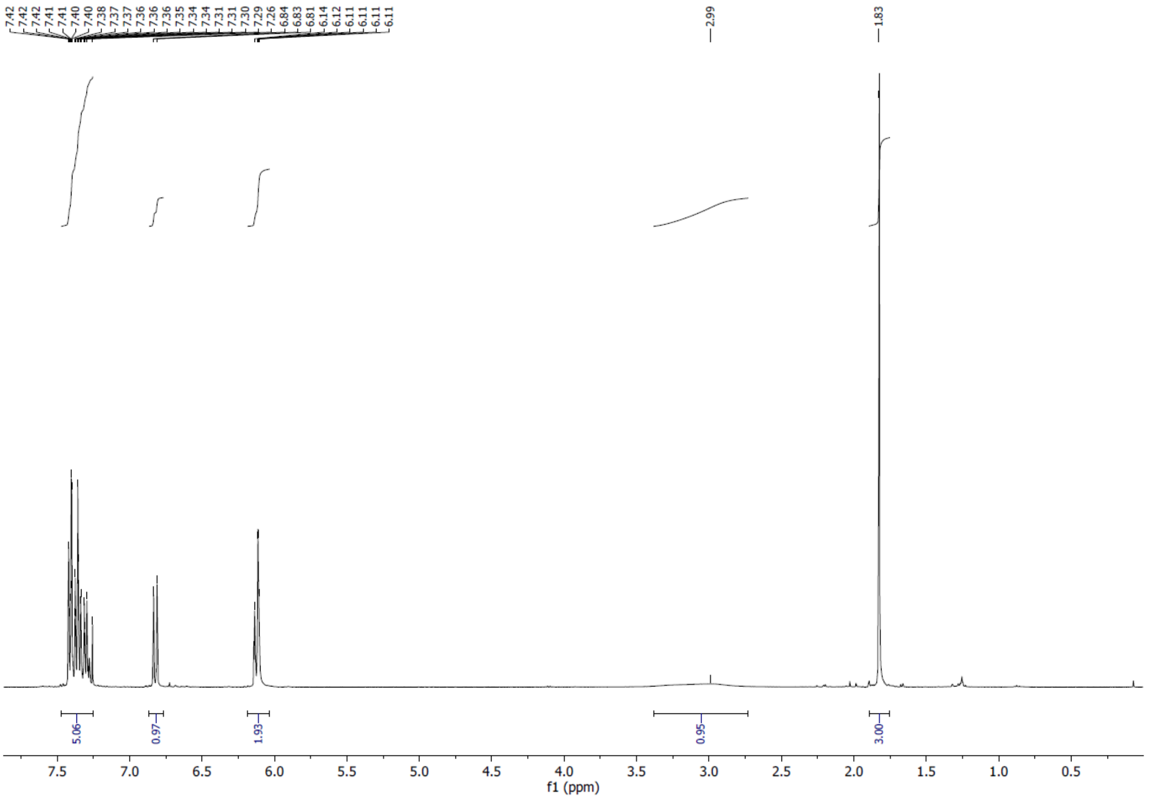

Figure S22. ^{1}H NMR spectra (400 MHz, $CDCl_3$) of 4-hydroxy-2-methyl-4-phenyl-2,5-cyclohexadienone (10)

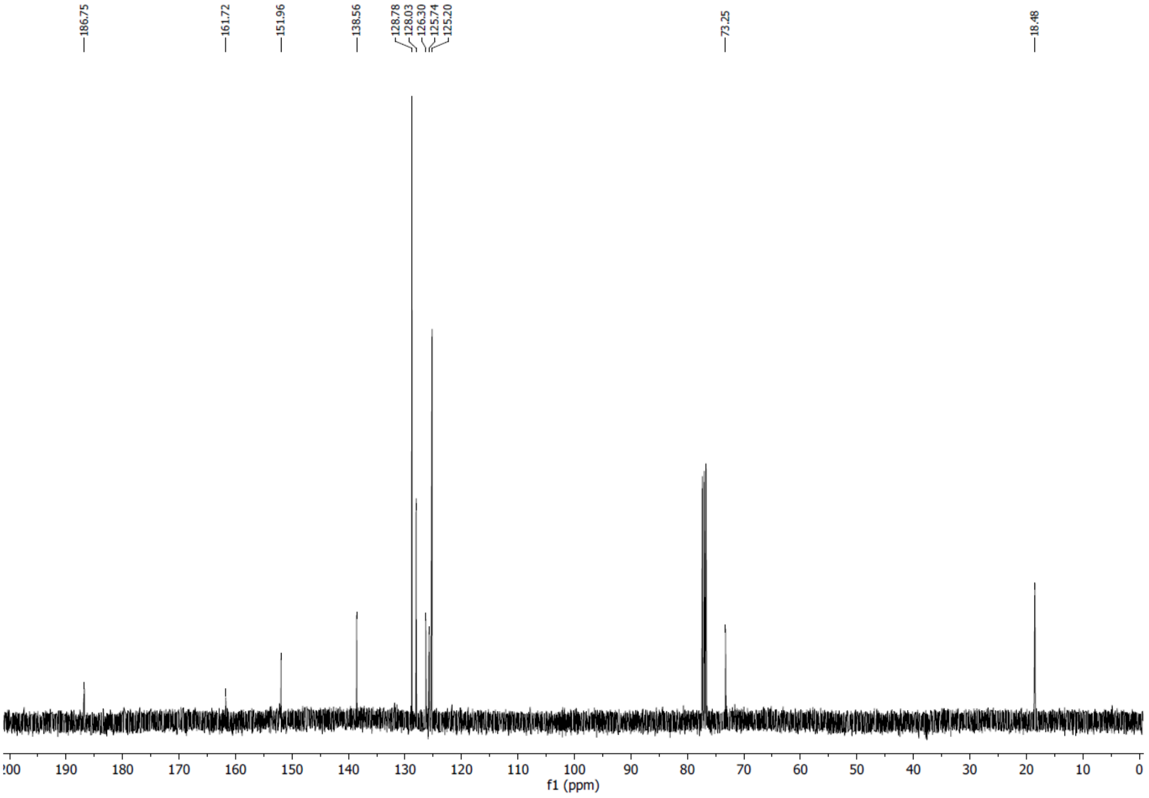

Figure S23. ^{13}C NMR spectra (100 MHz, $CDCl_3$) of 4-hydroxy-2-methyl-4-phenyl-2,5-cyclohexadienone (10)

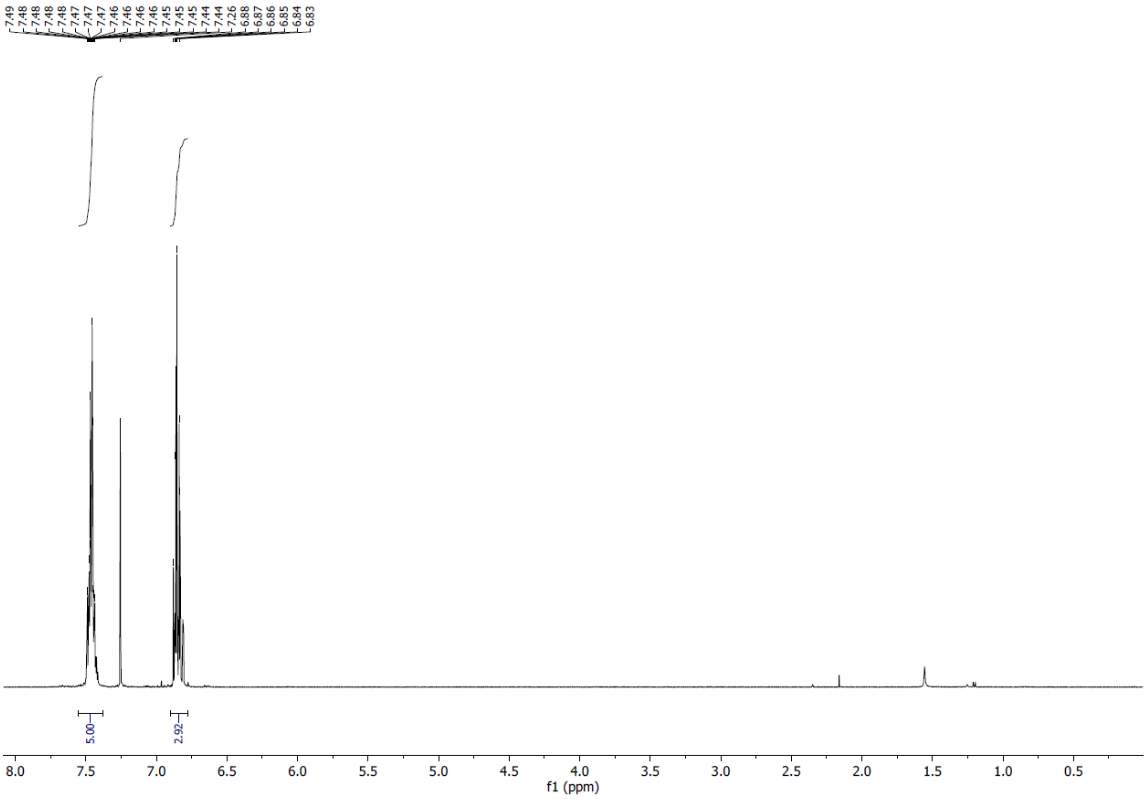

Figure S24. ^{1}H NMR spectra (400 MHz, $CDCl_3$) of 2-phenyl-1,4-benzoquinone (15)

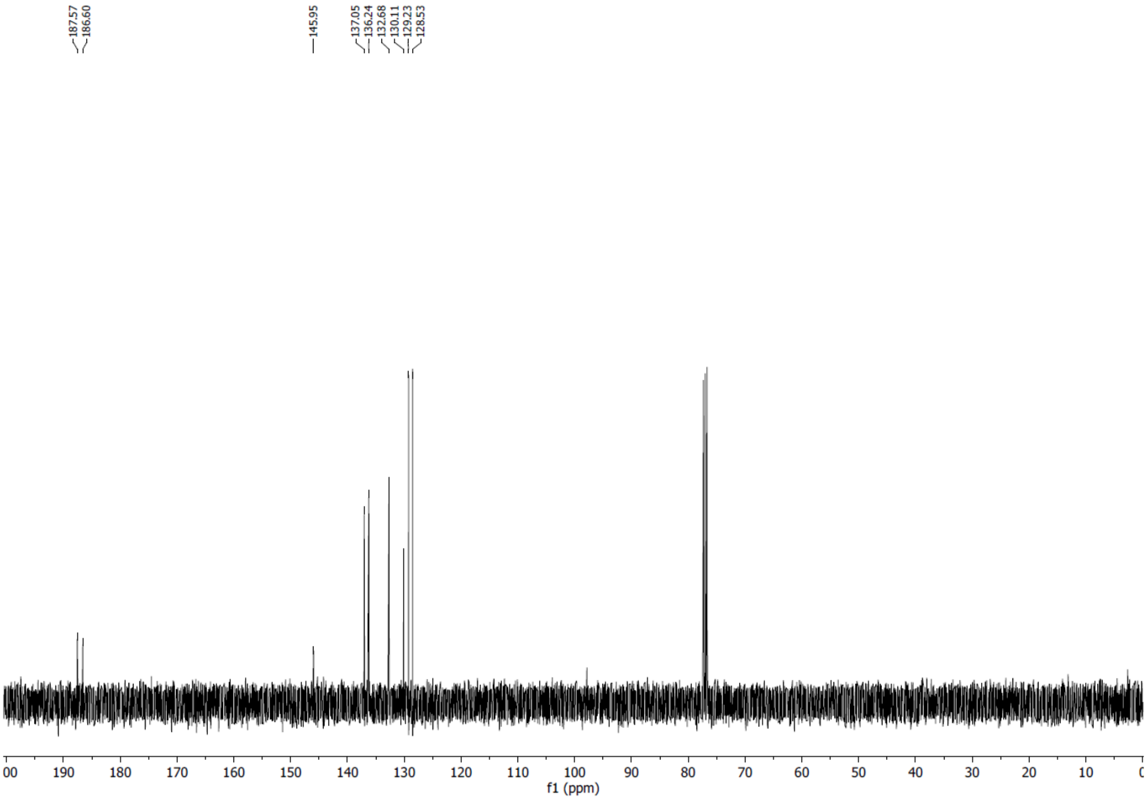

Figure S25. ^{13}C NMR spectra (100 MHz, $CDCl_3$) of 2-phenyl-1,4-benzoquinone (15)
